# An Evaluation of the Nutritional and Promotional Profile of Commercial Foods for Infants and Toddlers in the United States

**DOI:** 10.3390/nu16162782

**Published:** 2024-08-21

**Authors:** Daisy H. Coyle, Maria Shahid, Kiana Parkins, Monica Hu, Marina Padovan, Elizabeth K. Dunford

**Affiliations:** 1 The George Institute for Global Health, University of New South Wales, Level 18, International Towers 3300 Barangaroo Ave, Barangaroo, Sydney, NSW 2000, Australiaedunford@georgeinstitute.org.au (E.K.D.); 2 School of Public Health, University of California, Berkeley, CA 94704, USA; 3 Nutrition in Foodservice Research Center, Health Sciences Center, Federal University of Santa Catarina, Florianópolis 88040-900, SC, Brazil; 4 Department of Nutrition, Gillings Global School of Public Health, The University of North Carolina at Chapel Hill, Chapel Hill, NC 27599, USA

**Keywords:** baby food, nutrient profiling, marketing, claims, promotion, infants, toddlers

## Abstract

Despite growing concerns over the increasing popularity and health impact of commercial foods for infants and toddlers, no nutrition or promotional guidelines currently exist for the United States. In 2022, the WHO Regional Office for Europe published a nutrient and promotion profile model (NPPM) to provide guidance and regulation for commercially produced infant and toddler foods. This study assessed the nutritional and promotional profile of infant and toddler foods (6–36 months of age) collected from the top 10 grocery chains in 2023. Products were assessed against the WHO NPPM nutritional and promotional requirements. The type and number of claims across packaging type were also assessed. Of the 651 products examined, 60% failed to meet the nutritional requirements of the NPPM, and 0% met the promotional requirements. Almost 100% of products had at least 1 claim on-pack that was prohibited under the NPPM, with some products displaying up to 11 prohibited claims. Snack-size packages had the lowest compliance with nutrient requirements. These findings highlight that urgent work is needed to improve the nutritional quality of commercially produced infant and toddler foods in the United States. The high use of prohibited claims also suggests the need to regulate the type and number of claims allowed on-pack.

## 1. Introduction

Early childhood nutrition, particularly during the first 1000 days, is vital for healthy growth and development and for preventing overweight and obesity. Taste preferences and dietary habits are also formed during this critical stage of life, behaviors which often persist into adulthood [1,2]. Recognizing the need to optimize nutrition during these formative years, various governments and international organizations have developed dietary and feeding guidelines to provide guidance on what constitutes a healthy diet. These guidelines, such as those from the US Department of Agriculture (USDA) and the World Health Organization (WHO), recommend exclusive breastfeeding until six months of age with the introduction of appropriate solid foods at six months with continued breastfeeding up to two years [3,4]. The USDA guidelines [3] also recommend against feeding infants foods with added sugars and those high in sodium. Despite these recommendations, research consistently demonstrates that infants and toddlers across the world often fail to meet these recommendations, including in the United States [5,6,7].

It is now widely acknowledged that the food environment plays a significant role in influencing food choices and shaping dietary intakes [8]. Over recent decades, there has been a huge rise in the availability and popularity of processed, ready-to-eat food products for infants and young children [9]. A growing concern internationally is the suitability of many of these commercial foods, with studies conducted globally, including in the United States, showing that these products frequently contain high levels of added sugars, salt, and saturated fats [10,11]. As early childhood is an important period for shaping healthy eating habits, frequent consumption of these products may lead to long-term unhealthy dietary patterns and increase the chronic disease risk into adulthood [12].

In addition to concerns related to the healthiness of commercially produced infant and toddler foods, concerns about the use of health and nutrition content claims and wellness messaging on these products is growing. Infant and toddler foods are often extensively promoted with health and nutrition claims that make it difficult for parents and carers to assess their healthiness accurately [13]. The use of claims is frequently misleading, suggesting products are “healthy”, “convenient”, and “appropriate for child development” [14]. These promotional claims are often used by manufacturers to distract consumers from a product’s poor nutritional profile [9,15]. Research has also demonstrated that health and nutrition content claims are commonly used on products that contain nutrients of concern, such as sugars [16]. These claims are used to downplay or omit the mention of unhealthy nutrients, and instead highlight other aspects of a product, like “gluten-free” or “organic”, creating a “health halo” effect that can further mislead consumers into believing such products to be healthy [17].

In response to the need for better guidance and regulation for commercially produced infant and toddler food products, the WHO Regional Office for Europe published a nutrient and promotion profile model (NPPM) in 2022 [18]. The NPPM is designed to support policy changes in the early childhood foods sector to ensure commercial foods are of a high nutritional quality and are promoted appropriately toward young children aged 6–36 months. Despite growing concerns over the increasing popularity and health impact of the commercial foods for infants and toddlers that are available for sale, no nutrition or promotional guidelines currently exist for the United States, and no studies have yet examined the nutrition composition, labeling, and marketing of these products according to this gold standard [19].

The primary aim of this study was to assess how well commercial infant and toddler foods available for sale in grocery stores (online and in-store) in the United States in 2023 comply with the WHO NPPM nutritional standards. The secondary aim was to assess the overall prevalence of promotional claims displayed on product packaging and assess compliance of the claims with the requirements outlined in the WHO NPPM.

## 2. Materials and Methods

### 2.1. Data Source

The dataset for analysis comprised 669 commercial infant and toddler food products available in the United States in 2023. Researchers visited one location for 8 of the top 10 grocery store chains in the US (Walmart, Kroger, Costco, Ahold Delhaize, Publix, Sam’s Club, Target, and Aldi) in Raleigh, North Carolina, between March and May 2023. Two of the top ten grocery store retailer locations were not located in North Carolina, and as such, the websites for these two retailers were used to collect data (H-E-B and Safeway). Photos of all available products in the “baby” aisle (in-store) or under the “baby” tab (online) were collected. The George Institute’s FoodSwitch content management system was used to enter data captured from product photos [20]. The information extracted from FoodSwitch for analysis included manufacturer name, brand name, product description, ingredients, all health- and nutrition-related claims on product packaging, and nutrient information per serving and per 100 g (calories, protein, total fat, saturated fat, total sugars, added sugars, sodium).

### 2.2. Inclusion and Exclusion Criteria and Categorization

Infant formulas, fortified milk, and oral electrolytes were not included because the U.S. Food and Drug Administration regulates these products separately [21]. Following a previously published method [22], only products available in the baby food section of the grocery store were included. This meant yogurts located in the fridge section, drinks section, or under the “dairy, eggs, and fridge” tab online were not included. Finally, duplicate products were excluded, i.e., the same product in the same package size.

### 2.3. Food Categorization

Using the WHO NPPM taxonomy, each product was coded into one of eight broad food categories: (1) Dry cereals and starches; (2) Dairy foods; (3) Fruit and vegetable purées/smoothies and fruit desserts; (4) Savory meals/meal components: combinations of starches, vegetables, dairy, and/or traditional proteins; (5) Snacks and finger foods; (6) Ingredients; (7) Confectionery; and (8) Drinks. Within each broad category, products were further broken down into subcategories (Table S1). Each product was then assessed against the relevant nutrient composition standards for its subcategory.

### 2.4. The WHO Nutrient Profile and Promotion Model

This paper assessed the healthiness of the foods and beverages targeted toward infants and toddlers available for sale in the United States by comparing the nutrition composition against the WHO NPPM benchmarks. Benchmarks are set for different food categories according to their nutritional composition (e.g., energy density, sodium, etc.). Nutrient composition criteria can be seen in Table S1. Additionally, the WHO NPPM serves to restrict the marketing of inappropriate foods designed for infants and young children for health reasons through either mandatory or voluntary policies (Table S2). The NPPM provides a detailed list of claims that are considered “prohibited” and those that are allowed under the model. Claims coded as “allowed” in this project were those related to allergens, religious claims, or vegetarian/vegan claims. All remaining claims were coded as “prohibited”. To further examine the use of claims, each claim was placed into 1 of 14 high-level claim categories (allergen-related claims, cooking method claims, nutrition content claims, general health and nutrition claims, health claims, religious claims, safety and environment claims, serving-based claims, marketing claims, specific ingredient claims, texture-based claims, vegetarian/vegan claims, other dietary claims, and miscellaneous claims), and then into relevant subcategories (Table S3).

### 2.5. Statistical Analysis

Statistical analyses were undertaken using Stata V18. For each WHO subcategory, we assessed the number and proportion of products compliant with each part of the WHO NPPM nutritional and promotional criteria. In addition, the proportion of products displaying prohibited claims was examined (overall, by claim type, and by packaging type), as well as the mean and range of the number of claims reported on product packaging. Sales data from Euromonitor Passport [23] were used to demonstrate changes in the sales of infant and toddler foods in the United States by packaging type between 2010 and 2023.

## 3. Results

After removing duplicate products (*n* = 18), a total of 651 products could be mapped to a WHO subcategory and were included in the analysis. The number of products within each WHO subcategory ranged from *n* = 0 (for both “fruit snacks” and “dairy”) to *n* = 359 products (“fruit-containing purees, smoothies/desserts”). A total of 31 products belonged to the “confectionery” category and were therefore not included in the nutrient composition analysis, given that the WHO NPPM does not consider these products to be healthy (compliant) regardless of their nutrition profile. Out of these 651 products examined, 308 (47.3%) were pouches and a further 25.7% (*n* = 167) were ready-to-eat (RTE) jars, tubs, and containers (Table S4).

### 3.1. Compliance with Nutrient Composition and Front-of-Pack Labeling Criteria

Overall, 43.1% of the products (*n* = 267) were compliant with all of the relevant nutrition composition criteria under the WHO NPPM (Table 1). The lowest compliance among all products was found for “protein content” (29.6%) and the highest for “total fat” (92.7%). Overall, compliance by subcategory ranged from 0% for “savory meals/meal components without protein or cheese”, “dry or semi-dry snacks and finger foods”, and “ingredients” to 68.8% for “vegetable-only purees/smoothies/desserts”. There was a wide range of compliance in relation to protein content, from 3.3% for “dry or semi-dry snacks and finger foods” up to 100% for some of the “savory meal/meal components” products. Only 55.6% of products were compliant with the total sugar recommendations, with compliance lowest for “dry or semi-dry snacks and finger foods” (53.3%), “savory meals/meal components without protein or cheese” (53.5%), and “savory meals/meal components food with protein source not named first” (53.5%). Of note, 73.8% of “dry or semi-dry snacks and finger foods” contained added free sugar or sweetener. Only 57.5% (*n* = 374) of the products made an appropriate age-label claim. There was considerable variation in the use of age labels, with compliance ranging from 37.5% for “dry or powdered cereal/starch” up to 100% for “ingredients” and “savory meals/meal components with cheese named but no protein” (Table S4). A total of *n* = 18 products had an age label that reported the product to be suitable from 4+ months. While the WHO NPPM states that age labels should specify age in years or months, many products implied age through vague descriptions such as “sitter”, “tots”, “crawling baby”, or “toddler”. No products displayed a front-of-pack high-sugar flag as per the NPPM recommendation (Table S4).

### 3.2. Compliance with Promotional Composition Criteria

Overall, 0% of the products were compliant with all of the relevant promotional requirements (Table 2). This was due to 0% of the products meeting the “ingredient list clarity” requirement, as no products specified the weight (%) of ingredients and 0% of the products met the promotion and protection of breastfeeding criteria. This was followed closely by “no prohibited claims”, with only 0.6% meeting the requirement not to display these claims on the pack. Only 27.9% (*n* = 86) of the products displayed instructions to consume pouches by squeezing onto a spoon or bowl, and 69.8% (*n* = 60) of these products also stated that children can enjoy the product straight from the pouch.

Only 72.7% of the products had a product name that accurately reflected the order of ingredients on the ingredients list. There was a broad range of compliance observed, ranging from 21.3% for “dry or semi-dry snacks and finger foods” to 100% for “ingredients”, “savory meals/meal components with protein source named first”, and “savory meals/meal components with only protein named”. Lastly, 100% of the relevant products provided suitable preparation instructions, i.e., liquids used to reconstitute foods should not contain added sodium or free sugar, with all these products stating the products should be mixed only with water, formula, or breastmilk.

### 3.3. Frequency and Type of Claims On-Pack

**Figure 1:**
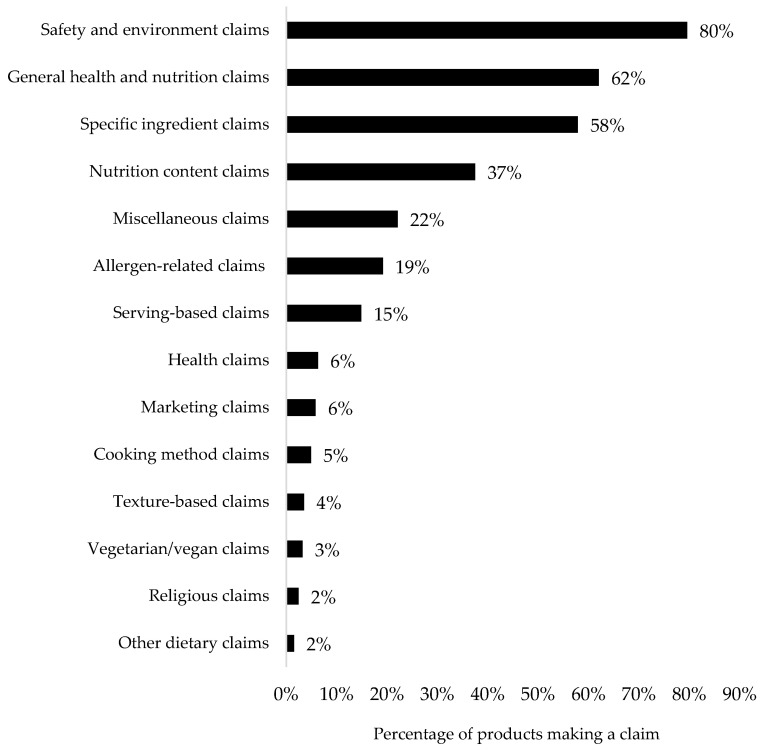
Frequency of claims use by type.

The mean number of nutrition and health-related claims per product was 4.7 (range: 1 to 13) (Table 3), with the majority being prohibited claims, appearing on-pack up to 11 times per product. “Savory meals/meal components with cheese”, “dry or semi-dry snacks and finger foods”, and “confectionery” had the highest number of claims overall (6.9 claims, 6.1 claims, and 6.1 claims, respectively). “Savory meals/meal components with cheese” and “Savory meals/meal components without protein and cheese” had the highest use of prohibited claims, on average. Of all 14 high-level categories of claims examined, the 3 that appeared most on infant and toddler food packages were safety and environment claims (79.6% of all products), primarily driven by “non-GM” claims (69.9%), “no BPA” claims (37.2%), and “no pesticides” claims (4.6%) (Figure 1). This was followed by general health and nutrition claims that were present on 62.1% of products overall. The most common general health and nutrition claims were “organic” claims (59.3%), “weaning” claims (e.g., baby-led-friendly) (3.4%), and “wholefoods” claims (3.2%). The third most common type of claim was specific ingredient claims, which were present on 62.1% of products overall. The most common specific ingredient claims on-pack were “no artificial colors and/or flavors” claim (25.0%), “no preservatives” claim (11.5%), and “no added sweeteners” claim (6.3%) (Figure 1).

### 3.4. Nutrition Composition, Promotional Criteria, and Claims Use by Packaging Type

**Figure 2:**
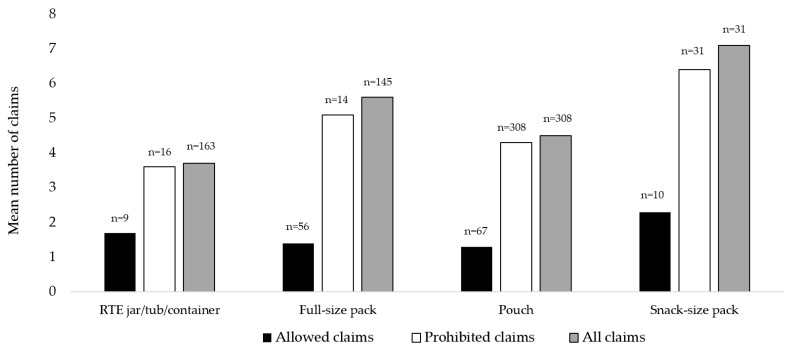
Claims use by packing type.

Overall, the nutrition composition compliance by packaging type ranged from 0.0% for snack-size packs to 56.8% for pouches (Table S5). For snack-size packs, zero products met protein requirements, 90.3% failed energy density requirements, 87.1% failed total sugar requirements, and 71.0% were not compliant with the requirement not to contain added free sugar or sweetener. RTE jars/tubs/containers had the lowest compliance with age labels (47.3%), followed by pouches (57.1%). Regarding compliance with promotion criteria, all packaging types had very low compliance with the “no prohibited claims” requirement, and all packaging types failed the “ingredient list clarity” requirement. Compliance with the “product name clarity” requirement ranged from 35.2% for full-size packs to 89.9% for RTE jar/tubs/containers. With respect to claims, all packaging types had a high use of claims, ranging from a mean of 3.7 claims per pack for RTE jar/tubs/containers to 7.1 for snack-size packs. Snack-size packs also had the highest use of prohibited claims, with 6.4 claims on-pack, on average (Figure 2). The most common claims on snack-size products included “organic” (90.3% of all products), “non-GM” (71.0%), and “no BPA” (32.3%). The most common claims on full-size packs included “non-GM” (77.2%), followed by “no artificial colors and/or flavors” (52.4%), and “organic” (49.7%). The most common claims on pouches were “organic” (76.0%), “non-GM” (61.7%), and “no BPA” (53.6%), and the most common claims on RTE jar/tub/containers were “non-GM” (74.3%), followed by “organic” (31.7%), and “no BPA” (31.1%). A huge increase in the proportion of sales of pouch products (and a concurrent decrease in RTE tubs/jars) was observed, from 6% in 2010 to 60% in 2023 (Figure S1), representing a 900% increase over a 13-year period.

## 4. Discussion

This study of 651 commercially produced infant and toddler foods available in the United States found that no products met international front-of-pack and promotional standards set by the WHO, and nearly 60% failed to meet the nutritional composition standards. Although compliance varied across subcategories, overall performance was poor, particularly for total sugar and protein content. Compliance with WHO standards for total sugar was particularly low snack-size packs, with fewer than 15% meeting the recommendations. Concerningly, many products made prohibited claims, with some having up to 11 prohibited claims per pack. The fact that all products failed to meet international standards highlights the urgent need for policymakers to regulate this sector to ensure that more complementary infant and toddler foods in the United States are suitable for children aged 6–36 months.

While there was variation in compliance with the WHO NPPM’s nutrition criteria, compliance was generally low across nearly all nutrients. The lowest compliance was found for total protein and total sugar, with over 70% of products failing to meet protein requirements and 44% exceeding total sugar recommendations. Additionally, a quarter of products failed to meet energy density requirements and contained added free sugar or sweeteners, and one-fifth failed to meet sodium requirements. The frequent use of free sugars and the high levels of sugar in infant and toddler food products in the United States is concerning, given that excess sugar consumption is a primary cause of obesity and related diseases, including diabetes, heart disease, and some cancers [24]. In light of the known health risks and the rapid growth of the commercial infant and toddler food market in the United States and globally [25], policymakers should consider setting limits on harmful sugars in these products and/or restricting the use of added free sugars and sweeteners. Nutrition composition requirements should also be considered for energy, protein, and sodium, given that compliance was low across many food categories.

Our analysis also identified specific concerns for convenience-style infant and toddler foods, particularly snack and finger foods and pouches. Snack and finger foods, such as fruit bars, cereal bars, and puffed snacks, made up nearly 20% of products available for purchase in 2023 yet had some of the lowest compliance rates across the WHO’s nutrition and promotional criteria. These foods contained low levels of protein and high levels of energy, sodium, and sugar and frequently contained added free sugars and sweeteners. Regarding pouches, our analysis, consistent with previous research [26], showed that these products have experienced a substantial 900% growth in the market over the past decade and now dominate the market, representing close to 50% of all products available for purchase in 2023. Concerningly, only 69% of pouches met international recommendations for total sugar. This finding is in line with existing research, which has shown that pouches contain higher levels of sugar compared with other packaging types in the infant and toddler food sector [27]. It is, therefore, unsurprising that other research has also shown that pouches are responsible for half of all sugar consumed from commercial infant and toddler foods [28]. Together, pouches and snack foods make up the vast majority of the market and are likely to continue increasing in popularity as parents lean toward these products over homemade foods due to busy lifestyles, rising birthrates, and a growing number of women in the workforce [25]. As such, policymakers should prioritize policies that regulate the nutrition composition and promotion of these products, which are not only unhealthier than their counterparts but also dominate the market in the United States.

All but four products failed to meet the WHO’s promotional criteria regarding prohibited claims. While only a small portion displayed allowed claims (e.g., “kosher”, “vegan”, “nut-free”), almost all products made at least one prohibited claim on-pack. On average, products displayed these claims up to 4 times, with some products displaying up to 11 different claims on a single package. Interestingly, safety and environmental claims were the most common, likely reflecting manufacturers responding to a potential interest among US parents and caregivers about genetically modified and BPA-free foods, along with organic. However, these claims were also often made in combination with nutrient and ingredient claims, which highlights that most food manufacturers in the United States use a range of claim techniques to encourage parents to buy their products. This extensive use of claims is consistent with prior research from Australia and the United Kingdom [14] and is of great concern. Claims are highly influential on infant and toddler foods; they not only boost a product’s appeal but also influence parental perceptions, often increasing the perceived healthiness of a product [29,30]. Given the very high prevalence of claims on US infant and toddler foods and their strong influence on consumer purchases, it is important that policymakers take action to prevent manufacturers from placing such influential promotional messages on foods intended for young children.

Consistent with prior research from Australia and the United Kingdom [14,31], one-third of infant and toddler foods had a misleading product name that did not reflect the order of the ingredients on the ingredients list. Compliance was particularly low for snacks and finger foods, where many products make reference to fruit or vegetables in the product name but primarily contain flour or other starches, with fruit and vegetable ingredients used in much smaller quantities (i.e., further down the ingredients list). Compliance with recommendations against consuming pouches directly from the spout was also low. Around 70% of products failed to warn against allowing children to feed directly from the spout, and those that did include a warning frequently also stated that pouches can be enjoyed via the spout as a second option. Given that research has shown that 65% of children who regularly consume pouches do so by sucking directly from the pouch [32], there is a strong need for clearer guidance on how these products should be consumed. Conflicting information around pouch use should be prohibited, as it likely weakens the message that these products should not be sucked directly. Lastly, despite the Center for Disease Control and Prevention [33] advising that solid foods should be introduced from six months of age, we identified a number of products that were explicitly marketed as suitable for children under this age or implicitly marketed as suitable through the use of terms such as “supported sitter”, “1st foods”, “sitting baby”. The availability of such products on the market can lead parents and caregivers to believe they should be introducing solid foods at an earlier age than is recommended, and therefore, the use of appropriate age labels should also be considered as part of the regulatory priorities for this sector [34].

The strengths of this analysis include the comprehensive dataset used, with products collected from the top 10 grocery stores in the US. A limitation of this study was that although we included a large representative dataset in the analysis, we did not have access to sales data for each product, and so it is not known whether consumers are purchasing more products that do not meet the requirements of the NPPM. Future research would benefit from linking these data with sales data to better understand what types of infant and toddler food products consumers are purchasing. Another limitation of this study was that the WHO NPPM was developed for the European region, and therefore is not necessarily 100% applicable to the US infant and toddler foods market. However, results from this study should demonstrate that there is a need for better regulation and guidance in the infant and toddler foods market in the United States.

## 5. Conclusions

This study found that no commercially produced infant and toddler food products available for purchase from the top 10 grocery store retailers in the United States met international standards for nutrition and product promotion. These findings should serve as a wake-up call for policymakers. The prolific use of prohibited claims demonstrates the need to regulate the type and number of claims that can be used on product packaging, ensuring caregivers are not misled by the deceptive labeling that is currently used. The study also found that pouches are the fastest growing packaging type in the infant sector, with a 900% increase in sales between 2010 and 2023, and that these products contain high levels of sugar and use claims frequently. Consequently, a reduction in the use of promotional claims, and in the sugar content and energy content (particularly for pouches), should form the basis of future policies in the infant and toddler food sector for the United States.

## Figures and Tables

**Table 1:** Compliance of commercial infant and toddler food products in the United States with the WHO NPPM nutritional criteria, by food category.

Food Category	Subcategory	Total Number and Percentage of Compliant Products, n (%)						
		Energy Density (kcal/100 g)	Sodium (mg/100 kcal)	Total Sugar (% Energy)	Added Free Sugar or Sweetener	Total Protein (g/100 g kcal)	Total Fat (g/100 kcal)	Compliance with All
Dry cereals and starches	Dry or powdered cereal/starch (*n* = 16)	16 (100.0%)	16 (100.0%)	-	9 (56.2%)	-	16 (100.0%)	9 (56.2%)
Dairy	Dairy (*n* = 0)	-	-	-	-	-	-	-
Fruit and vegetable purees/smoothies/fruit desserts	Fruit-containing products (*n* = 359)	272 (75.8%)	340 (94.7%)	-	309 (86.1%)	-	359 (100.0%)	218 (60.7%)
	Vegetable-only products (*n* = 48)	-	37 (77.1%)	-	48 (100.0%)	-	44 (91.7%)	33 (68.8%)
Savory meals/meal components	Food without protein or cheese named (*n* = 15)	12 (80.0%)	10 (66.7%)	8 (53.3%)	15 (100.0%)	6 (40.0%)	13 (86.7%)	0 (0.0%)
	Food with cheese named but no protein (*n* = 9)	9 (100.0%)	3 (33.3%)	6 (66.7%)	6 (66.7%)	5 (55.6%)	9 (100.0%)	1 (11.1%)
	Food with protein source not named first (*n* = 43)	39 (90.7%)	19 (44.2%)	23 (53.5%)	34 (79.1%)	36 (83.7%)	28 (65.1%)	2 (4.7%)
	Food with protein source named first (*n* = 4)	4 (100.0%)	4 (100.0%)	4 (100.0%)	4 (100.0%)	4 (100.0%)	3 (75.0%)	3 (75.0%)
	Protein source is only named food (*n* = 3)	3 (100.0%)	2 (66.7%)	3 (100.0%)	3 (100.0%)	3 (100.0%)	2 (66.7%)	1 (33.3%)
Snacks and finger foods	Fruit (*n* = 0)	NA	-	-	-	-	-	-
	Dry or semi-dry snacks and finger foods (*n* = 122)	78 (63.9%)	68 (55.7%)	65 (53.3%)	32 (26.2%)	4 (3.3%)	100 (82.0%)	0 (0.0%)
Ingredients	Ingredients (*n* = 1)	-	0 (0.0%)	-	0 (0.0%)	-	-	0 (0.0%)
Confectionery	Confectionery (*n* = 31)	-	-	-	-	-	-	-
Overall	*n* = 651	433 (75.8%)	499 (80.5%)	109 (55.6%)	460 (74.2%)	58 (29.6%)	574 (92.7%)	267 (43.1%)

**Table 2:** Compliance of commercial infant and toddler food products in the United States with the WHO NPPM labeling and promotion criteria, by food category.

Food Category	Subcategory	Total Number and Percentage of Compliant Products, n (%)					
		No Prohibited Claims	Product Name Clarity	Ingredient List Clarity	Instructions not to Consume via Pack Spout ^1^	Suitable Preparation Instructions	Promotion and Protection of Breastfeeding
Dry cereals and starches	Dry or powdered cereal/starch (*n* = 16)	0 (0.0%)	15 (93.8%)	0 (0.0%)	-	16 (100%)	0 (0%)
Dairy	Dairy (*n* = 0)	-	-	-	-	-	-
Fruit and vegetable purees/smoothies/fruit desserts	Fruit-containing products (*n* = 359)	0 (0.0%)	322 (89.7%)	0 (0.0%)	79 (29.5%)	-	0 (0%)
	Vegetable-only product (*n* = 48)	0 (0.0%)	47 (97.9%)	0 (0.0%)	2 (18.2%)	-	0 (0%)
Savory meals/meal components	Food without protein or cheese named (*n* = 15)	0 (0.0%)	13 (86.7%)	0 (0.0%)	2 (28.6%)	-	0 (0%)
	Food with cheese named but no protein (*n* = 9)	0 (0.0%)	4 (44.4%)	0 (0.0%)	-	-	0 (0%)
	Food with protein source not named first (*n* = 43)	0 (0.0%)	19 (44.2%)	0 (0.0%)	3 (13.6%)	-	0 (0%)
	Food with protein source named first (*n* = 4)	4 (100.0%)	4 (100.0%)	0 (0.0%)	-	-	0 (0%)
	Protein source is only named food (*n* = 3)	0 (0.0%)	3 (100.0%)	0 (0.0%)	-	-	0 (0%)
Snacks and finger foods	Fruit (*n* = 0)	-	-	-	-	-	-
	Dry or semi-dry snacks and finger foods (*n* = 122)	0 (0.0%)	26 (21.3%)	0 (0.0%)	-	-	0 (0%)
Ingredients	Ingredients (*n* = 1)	0 (0.0%)	1 (100.0%)	0 (0.0%)	-	-	0 (0%)
Confectionery	Confectionery (*n* = 31)	-	19 (61.3%)	-	-	-	-
Overall	*n* = 651	4 (0.6%)	473 (72.7%)	0 (0.0%)	86 (27.9%)	16 (100%)	0 (0%)

^1^ Four categories contained products that were relevant to the “instructions not to consume via spout” criteria: “fruit-containing purees/smoothies/fruit desserts” (*n* = 268), “vegetable-containing purees/smoothies/fruit desserts (*n* = 11), “savory meals without protein or cheese named” (*n* = 7), and “savory meals with protein source not named” (*n* = 22).

**Table 3:** Number and proportion of allowed and prohibited claims under the WHO NPPM, by subcategory.

Food Category	Subcategory	Allowed Claims			Prohibited Claims			All Claims		
		n (%) Displaying	Mean (SD) Number of Claims	Range	n (%) Displaying	Mean (SD) Number of Claims	Range	n (%) Displaying	Mean (SD) Number of Claims	Range
Dry cereals and starches	Dry or powdered cereal/starch (*n* = 16)	0 (0.0%)	-	-	16 (100.0%)	3.6 (1.9)	1–8	16 (100.0%)	3.6 (1.9)	1–8
Dairy	Dairy (*n* = 0)	-	-	-	-	-	-	-	-	-
Fruit and vegetable purees/smoothies/fruit desserts	Fruit-containing products (*n* = 359)	68 (18.9%)	1.3 (0.5)	1–3	359 (100.0%)	4.0 (1.9)	1–11	359 (100.0%)	4.3 (2.0)	1–13
	Vegetable-only products (*n* = 48)	3 (6.3%)	1.3 (0.6)	1–2	48 (100.0%)	3.3 (1.8)	1–9	48 (100.0%)	3.4 (1.9)	1–10
Savory meals/meal components	Food without protein or cheese named (*n* = 15)	4 (26.7%)	1.7 (0.6)	1–2	15 (100.0%)	5.7 (2.3)	2–10	15 (100.0%)	6.0 (2.7)	2–11
	Food with cheese named but no protein (*n* = 9)	2 (22.2%)	1.0 (0.0)	1–1	9 (100.0%)	6.7 (2.7)	3–10	9 (100.0%)	6.9 (3.0)	3–11
	Food with protein source not named first (*n* = 43)	2 (4.7%)	1.5 (0.7)	1–2	43 (100.0%)	3.9 (1.7)	1–9	43 (100.0%)	4.0 (1.8)	1–10
	Food with protein source named first (*n* = 4)	0 (0.0%)	-	-	0 (0.0%)	-	-	0 (0.0%)	-	-
	Protein source is only named food (*n* = 3)	0 (0.0%)	-	-	3 (100.0%)	1.0 (0.0)	1–1	3 (100.0%)	1.0 (0.0)	1–1
Snacks and finger foods	Fruit (*n* = 0)	-	-	-	-	-	-	-	-	-
	Dry or semi-dry snacks and finger foods (*n* = 122)	53 (43.4%)	1.4 (1.0)	1–6	122 (100.0%)	5.5 (2.1)	2–10	122 (100.0%)	6.1 (2.2)	2–13
Ingredients	Ingredients (*n* = 1)	0 (0.0%)	-	-	1 (100.0%)	5.0 (-)	5–5	1 (100.0%)	5.0 (-)	5–5
Confectionery	Confectionery (*n* = 31)	10 (32.3%)	1.1 (1.4)	1–3	31 (100.0%)	5.5 (1.7)	3–9	31 (100.0%)	6.1 (1.4)	4–9
Overall	*n* = 651	142 (21.8%)	1.4 (0.7)	1–6	647 (99.4%)	4.4 (2.1)	1–11	647 (99.4%)	4.7 (2.2)	1–13

## Data Availability

The data that support the findings of this study are available from The George Institute for Global Health. Restrictions apply to the availability of these data, which were used under license for this study.

## Supplementary Materials

The following supporting information can be downloaded at: https://www.mdpi.com/article/10.3390/nu16162782/s1, Table S1: WHO NPPM Part A: nutrient content requirements (adapted from WHO NPPM1); Table S2: WHO NPPM Part B: promotional messages (packets, labeling, and marketing) (adapted from WHO NPPM1); Table S3: Claims taxonomy for commercial infant and toddler food products in the United States; Table S4: Compliance of commercial food products in the United States with the WHO NPPM front-of-pack labeling criteria; Table S5: Compliance of commercial food products in the United States with the WHO NPPM labeling and promotion criteria, by packaging type; Figure S1: Change in sales of commercial infant and toddler foods in the United States by packaging type, 2010 to 2023.
