# Supplementary material for: An Evaluation of the Nutritional and Promotional Profile of Commercial Foods for Infants and Toddlers in the United States
Source: Nutrients. 2024 Aug 21;16(16):2782. doi: 10.3390/nu16162782 (PMC11357546; doi:10.3390/nu16162782)
Supplement: Supplementary file 1 [file nutrients-16-02782-s001.zip › nutrients-3140023-supplementary.pdf]

Supplementary Files

Table S1: WHO NPPM Part A: nutrient content requirements (adapted from WHO NPPM[1])

Food category	Subcategory	Content and labelling requirements						
		**Energy density (kcal/100g)**	**Sodium (mg/100 kcal)**	**Total sugar (% energy)**	**Added free sugar or sweetener**	**Total protein (g/100 kcal)**	**Total fat (g/100 kcal)**	**Fruit content (% weight)**
**Dry cereals and starches**	Dry or powdered cereal /starch	>=80 as eaten	<=50 as eaten	-	None	<=5.5g (if contains milk)	<=4.5g or <=3.3g (if eaten with milk)	<=100% dry weight
**Dairy**	Dairy-based foods, desserts and cereals	>=60	<=50 (100 if named cheese)	-	None	-	<=4.5g	<=5% (max. 2% dry)
**Fruit & vegetable purées/ smoothies and fruit desserts**	Fruit-containing product	>=60	<=50	-	None	-	<=4.5g	-
	Vegetable only product	<=25% added water	<=50	-	None	-	<=4.5g	None
**Savory meals/meal components: combinations of starches, vegetables, dairy and/ or traditional protein**	Food without protein or cheese named	>=60	<=50	<=15%	None	>=3g	<=4.5g	<=5% (max 2% dry)
	Food with cheese named but no protein	>=60	<=100	<=15%	None	>=3g	<=6g	<=5% (max 2% dry)
	Food with protein source not named first	>=60	<=50 (100 if named cheese)	<=15%	None	>=3g >=8%	<=4.5g	<=5% (max 2% dry)
	Food with protein source named first	>=60	<=50 (100 if named cheese)	<=15%	None	>=4g >=10%	<=6g	<=5% (max 2% dry)
	Protein source is only named food	>=60	<=50	<=15%	None	>=7g >=40%	<=6g	<=5% (max 2% dry)
**Snacks and finger foods**	Fruit	<=50 kcal per serve	<=50	-	None	-	<=4.5g	100%
	Dry or semi-dry snacks and finger foods	<=50 kcal per serve	<=50	<=15%	None	<=5.5g (if biscuit and contains milk)	<=4.5g	-
**Ingredients**	Ingredients	-	<=50	-	None	-	-	None
**Confectionery**	Confectionery	Not appropriate for promotion						

Table S2: WHO NPPM Part B: promotional messages (packets, labelling and marketing) (adapted from WHO NPPM[1])

Promotional requirement	Details and examples
**No compositional, nutritional, health or marketing claims**	No compositional, nutritional, health or marketing claims are permitted on packs or related marketing materials (promotional communications, websites, etc.). Note the following composition statements are permitted:<br>• statements relating to common allergens (such as containing or being "free from… [gluten, dairy/lactose, or nuts]" etc.)<br>• statements relating to religious or cultural requirements (such as "meat-free", "vegetarian", "contains meat", "Kosher", "Halal", etc.)<br>• descriptive words may be used within the ingredient list (such as "organic carrots" and "wholegrain wheat flour")
**Product name clarity**	The front-of-pack product name and legal product name must:<br>• clearly represent or name the main or largest ingredients, where appropriate, except when the largest ingredient is implied in the name (such as milk in porridge or rice in risotto);<br>• list ingredients in an appropriate order (to indicate decreasing proportional content); and<br>• indicate when fruit or vegetables (single or in combination) comprise the majority of the product by weight. Note that fruit or vegetables are considered to be the largest ingredient if the sum of all fruits or vegetables is the largest ingredient, and the front-of-pack name must indicate this<br>Note that all ingredients do not need to be listed in the product name
**Ingredient list clarity**	The ingredient list must clearly indicate the proportion (%) of:<br>• the largest single ingredient (including water/stock, except when used for rehydration of legumes/grains etc.)<br>• the amount of added water/stock (except when used for rehydration of legumes/grains etc.)<br>• the total or individual proportions of fresh or dried fruit<br>• the amount of fish, poultry, meat or other traditional source of protein
**Instructions not to consume soft foods via pack spout**	Ready-to-eat puréed foods sold in packs with a spout must include a clear statement to discourage caregivers from allowing infants and young children to suck the food directly via the spout, such as: "Infants and young children should not be allowed to suck directly from the pouch/container"
**Suitable preparation instructions**	Preparation instructions for dry cereals/starches, ingredients and meal components must state that the liquid used to reconstitute the product, or accompanying foods served, should have no added sodium or free sugar (including fruit juice)
**Promotion and protection of breastfeeding**	In relation to breast feeding:<br>• no cross-promotions are permitted between products that function as breastmilk substitutes, and commercially available complementary foods marketed as suitable for infants and young children > 6 months;<br>• all products must include a statement on the importance of continued breastfeeding for up to two years or beyond and the importance of not introducing complementary feeding before 6 months of age;<br>• no products should include any image, text or other representation that is likely to undermine or discourage breastfeeding, or that makes a comparison to breastmilk or that suggests that the product is nearly equivalent or superior to breastmilk;<br>• all products must state the suitable age of introduction (≥ 6 months);<br>• no products should include any image, text or other representation that might suggest use for infants under the age of 6 months (including references to milestones and stages); and<br>• no product should convey an endorsement or anything that may be construed as an endorsement by a professional or other body, unless this has been specifically approved by relevant national, regional, or international regulatory authorities.

Table S3: Claims taxonomy for commercial infant and toddler food products in the United States

Level 1	Level 2	Level 3	Allowed under WHO NPPM[1]
Allergens	Free from / No	Nuts	
		Grains	
		Rice	
		Gluten	
		Nuts	YES
		Peanut	
		Corn	
		Wheat	
	Made in a peanut free facility		
Cooking method	Baked / Simply baked		
	freeze-dried		
	Never fried		
	Not diluted with water		NO
	Oven baked		
	Heat treated after culturing		
	Cold pressure		
General health and nutrition	Organic / USDA organic / Organically grown		
	Premium / real / honest ingredients		NO
	Weaning		
	Whole food /whole foods / whole food clean eating		
Health claim	Calcium		
	Choline		
	Iron		
	Beta glutan		NO
	Vitamin A		
	Vitamin C		
	Vitamin E		
Marketing to children	Character on pack		NO
Miscellaneous claims	Great for you		
	Natural / Always natural / Naturally flavored / Natural flavors		
	Live active cultures		
	Brain supporting nutrients / Essential nutrients		
	Easily digestible		NO
	Prepared with ingredients only found in your kitchen		
	Family owned		
	Nourishing brain and body		
	Shelf-stable		
	Veggie power		

	Only / Just X ingredients		
	Veggie-first		
	Great taste		
	Simple / Select ingredients		
	Nothing	Artificial	
		Artificial added	
Nutrition content claim	Fortified	Iron	NO
	Grams of / mg of	Protein / Plant Protein	
		Omega 3 / Omega-3 fatty acids	
		Prebiotic fiber / fiber	
		Whole grains	
		Added sugars	
		Trans fat	
		Fat	
	%DV	Iron	
		Vitamin C / A	
		Potassium	
		Magnesium	
		Fiber	
	Rich in / High in	Vitamins/minerals	
		Antioxidant	
		Vitamin C	
	Source of / good source of	Calcium	
		Fiber	
		Iron	
		Omega-3	
		Protein	
		Vitamin B	
		Vitamin C	
	No / No added	Salt	
		Sugar	
	Un	Sweetened	
		Salted	
Religious	Kosher		YES
Safety/environment	Clean / Certified clean	Label	NO
		Field farming practices	
	Free from / does not contain / No / Non / Grown without	BPA	
		BPS	
		Phlalates	
		Chemicals	
		GMO/GM	
		Prohibited synthetic ingredients	

		Prohibited chemical pesticides	
		Toxic persistent pesticides	
		Prohibited chemical pesticides	
		Pesticides / Harmful pesticides	
	x% less	Plastic	
	Regeneratively farmed		
	Biodynamic		
	Carbon neutral<br>Carbon trust certified		
	Planetary		
	Healthy planet + healthy food		
	Made with renewable energy<br>Climate forward		
	No	Nutrition	
		Nutrition for baby	
		Plastic	
		Medicine	
Servings	Serving / servings of / Full serve /Full serving	Vegetables	NO
		Fruit	
		Fruit and vegetables	
		Superfoods / nutrient-dense superfoods	
	X types per serve		
	X Cup	Vegetables	
Specific ingredients	Contains / Made with / With / Crafted with	Real fruit / real fruit and vegetables	NO
		Whole grains	
		Yoghurt cultures	
		Free range / Pasture raised	
		Ancient grains	
		Real organic whole eggs	
		Grass-fed beef	
		Natural fruit and vitamins	
		Bone broth	
		Gelatin	
	Free from / does not contain / No / No added / Made without	Artificial colors / Synthetic colors	
		Artificial flavors	
		Concentrates	

		Artificial preservatives	
		Colors	
		Flavors	
		Hormones	
		Cane syrup	
		Trans fat	
		Salt	
		Preservatives	
		Sweeteners / artificial sweeteners	
		Hydrogenated oils	
		High fructose corn syrup	
		Industrial seed oils	
		Filler	
		Concentrates	
		Fillers	
		Preservatives	
	Flavored with	Real fruits / Real fruit and veggies	
	Sweetened with	Fruit juice	
	Fruit & Veggie blend		
Texture	Advanced		NO
	Gentle on gums		
	Melts in baby's mouth		
	Dissolves easily		
Vegan/vegetarian	Vegan / Vegan Friendly		YES
	Vegetarian		
Other dietary	Plant-based / plant-based protein / 100% plant-based nutrients / plant-powered		NO
	Paleo friendly		

Table S4: Compliance of commercial food products in the United States to the WHO NPPM[1] front-of-pack labelling criteria

Food category	Subcategory	Total number and percentage of compliant products, n(%)	
		Age label (months)	Front-of-pack high-sugar flag (% energy)
**Dry cereals and starches**	Dry or powdered cereal/starch (n = 16)	6 (37.5%)	0 (%)
**Dairy**	Dairy (n = 0)	N/A	N/A
**Fruit & vegetable purees/smoothies/fruit desserts**	Fruit-containing products (n = 359)	182 (50.7%)	0 (%)
	Vegetable only product (n = 48)	23 (47.9%)	0 (%)
**Savory meals/meal components**	Food without protein or cheese named (n = 15)	13 (86.7%)	0 (%)
	Food with cheese named but no protein (n = 9)	9 (100.0%)	0 (%)
	Food with protein source not named first (n = 43)	30 (69.8%)	0 (%)
	Food with protein source named first (n = 4)	0 (0.0%)	0 (%)
	Protein source is only named food (n = 3)	0 (0.0%)	0 (%)
**Snacks and finger foods**	Fruit (n = 0)	N/A	0 (%)
	Dry or semi-dry snacks and finger foods (n = 122)	85 (69.7%)	0 (%)
**Ingredients**	Ingredients (n = 1)	1 (100.0%)	0 (%)
**Confectionery**	Confectionery (n = 31)	N/A	0 (%)
**Overall**	**n = 651**	**374 (57.5%)**	**0 (%)**

Table S5: Compliance of commercial food products in the United States to the WHO NPPM labelling and promotion criteria, by packaging type

Packaging type	Total number and percentage of compliant products, n(%)						
	**Energy density (kcal/100g)**	**Sodium (mg/100kcal)**	**Total sugar (% energy)**	**Added free sugar or sweetener**	**Total protein (g/100g kcal)**	**Total fat (g/100 kcal)**	**Compliance with all**
RTE jar/tub/container (n = 167)	103 (79.8%)	134 (80.2%)	23 (52.3%)	138 (82.6%)	30 (68.2%)	162 (97.6%)	81 (48.5%)
Full-size pack (n = 145)	98 (84.5%)	75 (64.7%)	62 (66.0%)	35 (30.2%)	4 (4.3%)	93 (80.2%)	11 (9.5%)
Pouch (n = 308)	229 (77.1%)	275 (89.3%)	20 (69.0%)	278 (90.3%)	30 (68.2%)	290 (94.2%)	175 (56.8%)
Snack-size pack (n = 31)	3 (10.3%)	15 (51.7%)	4 (13.8%)	9 (31.0%)	0 (0.0%)	29 (100%)	0 (0.0%)
	**No prohibited claims**	**Product name clarity**	**Ingredient list clarity**	**Instructions not to consume via pack spout**	**Suitable preparation instructions**	**Promotion and protection of breastfeeding**	**Compliance with all**
RTE jar/tub/container (n = 167)	4 (4.2%)	150 (89.8%)	0 (0.0%)	N/A	N/A	0 (0.0%)	0 (0.0%)
Full-size pack (n = 145)	0 (0.0%)	51 (35.2%)	0 (0.0%)	N/A	16 (100%)	0 (0.0%)	0 (0.0%)
Pouch (n = 308)	0 (0.0%)	261 (84.7%)	0 (0.0%)	86 (27.9%)	N/A	0 (0.0%)	0 (0.0%)
Snack-size pack (n = 31)	0 (0.0%)	11 (35.5%)	0 (0.0%)	N/A	N/A	0 (0.0%)	0 (0.0%)

Figure S1: Change in sales of commercial infant and toddler foods in the United States by packaging type, 2010 to 2023

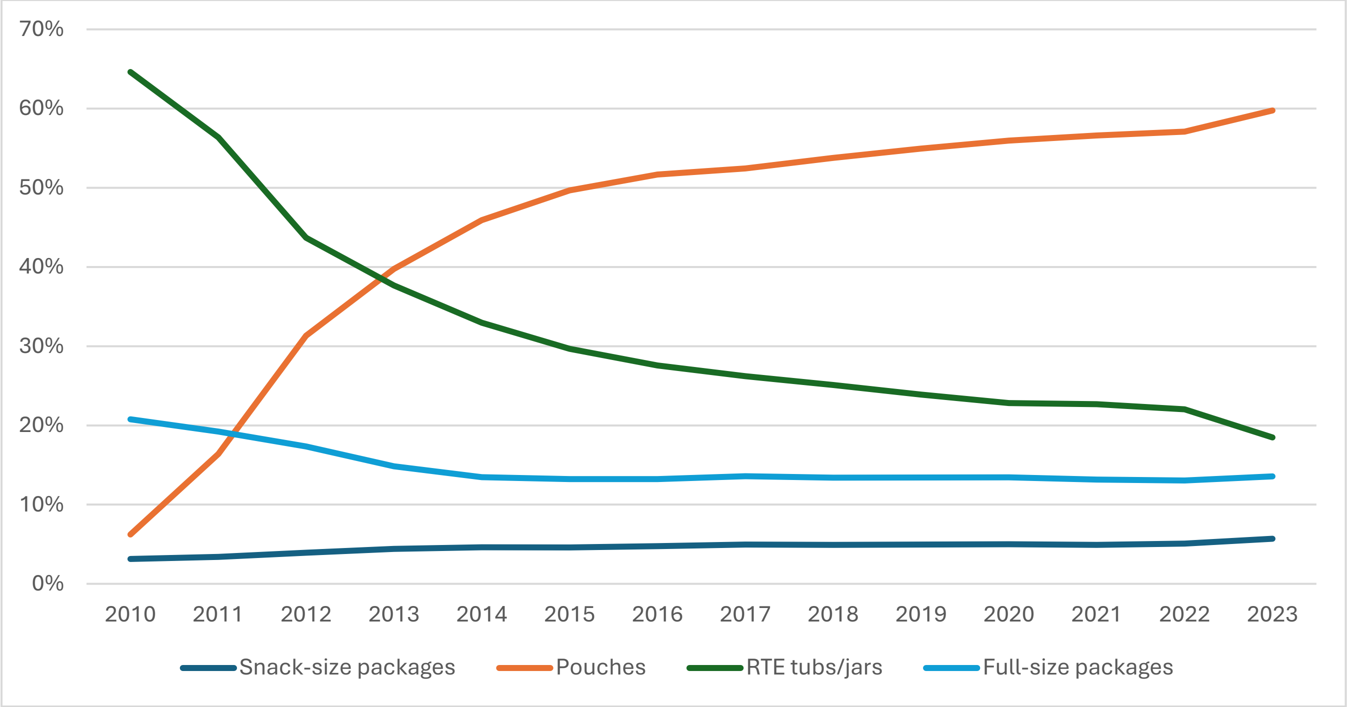

References

1. World Health Organization. Nutrient and Promotion Profile Model: Supporting appropriate promotion of food products for infants and young children 6–36 months in the WHO European Region, 2022.
